# Estimated mortality of the highly pathogenic avian influenza pandemic on northern gannets (*Morus bassanus*) in southwest Ireland

**DOI:** 10.1098/rsbl.2023.0090

**Published:** 2023-06-14

**Authors:** Oriol Giralt Paradell, Tiffany Goh, Dimitar Popov, Emer Rogan, Mark Jessopp

**Affiliations:** ^1^ School of Biological, Earth and Environmental Sciences, University College Cork, Enterprise Centre, Distillery Fields, Cork, Ireland T23 N73K; ^2^ MaREI Centre, Environmental Research Institute, University College Cork, Cork P43 C573, Ireland; ^3^ Green Balkans NGO, 1 Skopie street, 4000 Plovdiv, Bulgaria

**Keywords:** highly pathogenic avian influenza, northern gannet, southwest Ireland, aerial surveys, mortality

## Abstract

The 2022 highly pathogenic avian influenza (HPAI) outbreak that occurred in many European countries affected several seabird species. Among them, northern gannets (*Morus bassanus*) were particularly impacted. We conducted aerial surveys in waters around the two largest gannet colonies in southwest Ireland (Little Skellig and Bull Rock, together representing 87% of the national population) in September 2022. During surveys dead and alive northern gannets were counted on survey effort. A total of 184 dead gannets were recorded on survey effort, representing 3.74% of the total number of gannets recorded. We estimated the abundance of dead gannets in the surveyed area at 1526 (95% confidence intervals (CIs) 1450–1605) individuals. The percentage of dead gannets observed was used to estimate a minimum local population mortality of 3126 (95% CIs 2993–3260) individuals across both colonies. Aerial surveys provided key information on gannet mortality from HPAI at sea. The study provides the first estimate of gannet mortality in the two largest gannetries in Ireland.

## 1. Introduction

Since its emergence in poultry farms in Southeast Asia, highly pathogenic avian influenza (hereafter HPAI) outbreaks have mainly involved domestic birds [1,2]. However, infections have also been documented in wild birds [1], including seabirds [2]. In Europe, significant outbreaks, largely associated with poultry farming, were reported in 2016/2017, 2020/2021 and 2021/2022 [3]. However, the 2021/2022 outbreak saw the virus spread widely among wild bird populations, with seabird populations particularly affected. The route of transmission to seabirds is unknown, but may have occurred through scavenging species interacting with infected poultry and subsequently visiting seabird colonies. Due to breeding in dense terrestrial colonies and transmission of the virus occurring through contact with contaminated faeces [4], large numbers of terns, gulls and gannets were observed dead in colonies or washing up on beaches, with tests confirming the presence of HPAI H5 strain [5,6].

Europe holds approximately 83% of the world's northern gannet (*Morus bassanus*, Linnaeus 1758, hereafter gannet) population [7], with 52% of the European population occurring in the UK and Ireland [8,9]. HPAI was suspected in a number of UK colonies in June and on the island of Helgoland in the southeastern North Sea in early July 2022. As this is the first major outbreak of HPAI affecting gannet colonies at a large scale, information on the spread of the disease and the mechanisms that contributed to it is still being gathered and analysed. Recent reports and studies suggest a westward migration from central and northern Europe towards the UK and Ireland, where HPAI would have arrived at the end of summer 2022 [3,10]. The first case of HPAI in Irish gannet colonies was reported in August [11] and in September 2022, the authority responsible for testing in Ireland, the Department of Agriculture, Food and the Marine, had sufficient evidence for HPAI in wild birds that they stopped testing carcasses derived from the summer outbreak. Given the large numbers of dead gannets observed in colonies or washing up on shores, studies to assess the degree of impact at regional and population levels are vital. Here, we aim to provide an initial estimate of gannet mortality during the 2022 HPAI outbreak using aerial surveys to count dead birds surrounding Ireland's two largest gannetries.

## 2. Material and methods

Aerial surveys were conducted along the southwest coast of Ireland encompassing the two largest gannet colonies in the country, Little Skellig (35 294 breeding pairs) and Bull Rock (6388 breeding pairs), and surrounding waters used for foraging. These two colonies account for 87% of the country's breeding gannet population [9].

Surveys were conducted in September 2022 over an area of approximately 12 036 km^2^ using a twin-engine fixed wing aircraft equipped with bubble windows to afford observers an unrestricted view of the sea below the aircraft. Transects were spaced approximately 3.7 km apart (electronic supplementary material, figure SM1) and flown at a height of 75 m above sea level and a speed of approximately 185 km h^−1^ using strip-transect methodology [12] in target sea state of Beaufort less than or equal to 3 and greater than 2 km visibility. Two observers recorded all seabirds within 200 m either side of the aircraft along the transect lines noting species, number of birds and behaviour, with each sighting date/time linked to location using a Garmin GPS 60.

Gannets could be distinguished from other seabird species due to their larger size and coloration, and dead individuals were identified by posture; floating sideways with an extended neck and the wings not folded against their body, while live individuals were in flight or sitting on the water with the wings folded against the body.

Survey effort and density of gannets were calculated in QGIS 3.22.7 (http://www.qgis.org) using a 4 × 4 km grid across the study area. The total area surveyed per cell was estimated by multiplying the distance flown in each cell by the strip width (0.4 km if both observers were on effort, 0.2 km if one observer was on effort, 0 if both observers were off effort). The number of dead and alive gannets within each cell was counted and divided by the survey effort to obtain the density (individuals km^−2^) of dead and alive gannets. Estimated total abundance across the survey area was obtained by multiplying density by cell area and summing across all cells [13]. Poisson 95% confidence intervals (CIs) were calculated following the exact method:

$$\frac{\mathrm{qchiscq}(0.025,2\;\times \;x)}{2}\;\;\mathrm{and}\;\;\frac{\mathrm{qchiscq}(0.975,2\;\times \;(x+1))}{2}.$$

To assess the representativeness of the survey area in relation to the foraging ranges of gannets from Little Skellig and Bull Rock, tracks from 23 individuals (nine from Little Skellig and 14 from Bull Rock) previously published by Wakefield *et al.* [14] were used to calculate a 50% kernel utilization distribution (UD), representing the core foraging range, for each colony using the R Package Adehabitat HR [15]. Separate colonies UDs were merged in QGIS to obtain a combined representative foraging range for both colonies, and the percentage of overlap with the surveyed area was calculated. We made an initial estimate of the population level impact of HPAI in southwest Ireland calculated as

$$\begin{array}{rl} \mathrm{Estimated}\;\mathrm{population}\;\;\mathrm{mortality}\;(\mathrm{EPM})= & \mathrm{breeding}\;\mathrm{gannets}\\ \;\times \;\left(\frac{\mathrm{mean}\;\;\mathrm{dead}\;\mathrm{gannets}\;\mathrm{estimate}}{\mathrm{mean}\;\;\mathrm{alive}\;\;\mathrm{annets}\;\;\mathrm{estimate}}\right). \end{array}$$

Maximum and minimum estimates were calculated as

$$\begin{array}{rl} & \mathrm{Min}\;EPM=\mathrm{breeding}\;\;\mathrm{gannets}\\ & \;\times \left(\min\;\;\mathrm{dead}\;\mathrm{gannet}\;\mathrm{estimate}\;\times \frac{\max\;\mathrm{dead}\;\mathrm{gannet}\;\mathrm{estimate}\;}{\min\;\mathrm{alive}\;\mathrm{gannet}\;\mathrm{estimate}}\right), \end{array}$$

and

$$\begin{array}{rl} & \mathrm{Max}\;EPM=\mathrm{breeding}\;\mathrm{gannets}\\ & \;\times \left(\max\;\mathrm{dead}\;\mathrm{gannet}\;\mathrm{estimate}\;\times \frac{\min\;\mathrm{dead}\;\mathrm{gannet}\;\mathrm{estimate}\;}{\max\;\mathrm{alive}\;\mathrm{gannet}\;\mathrm{estimate}}\;\right). \end{array}$$

## 3. Results

**Figure 1.**
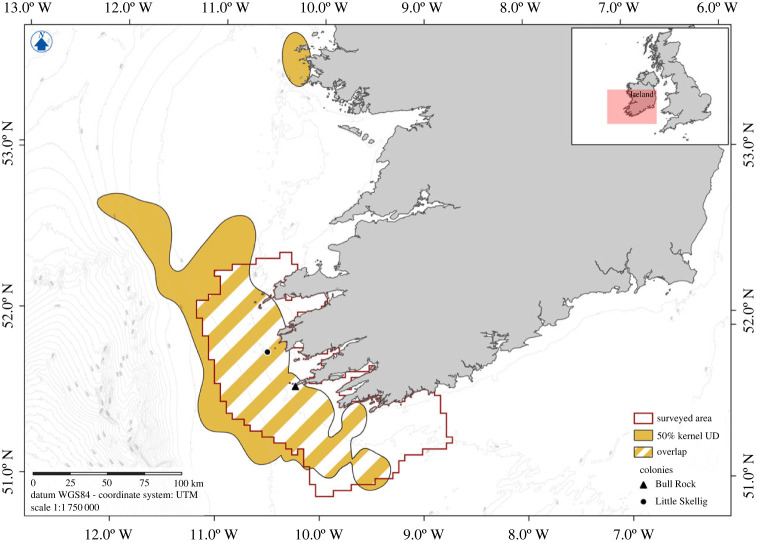
Overlap between the core foraging range and the surveyed area.

A total area of 12 036 km^2^ was surveyed with an average of 11% of grid cell area covered by observer effort (electronic supplementary material, figure SM2). Overlap of the 50% kernel UD denoting core gannet foraging areas within the survey area was high (62.6% of the survey area considered core foraging range, figure 1), indicating that the survey area was representative of the foraging range and therefore of the distribution of gannets at sea.

**Figure 2.**
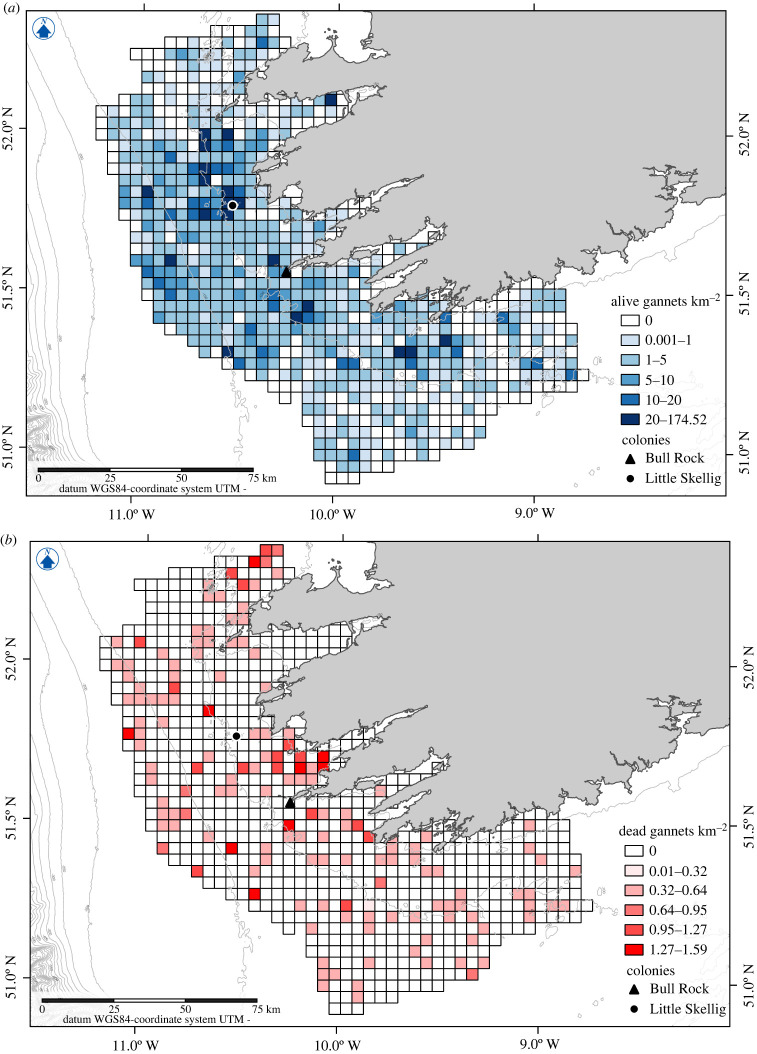
Densities of alive gannets (number of gannets km^−2^, (*a*)) and dead gannets (number of dead gannets km^−2^; right, (*b*)) in each cell.

One thousand nine hundred and seventy-three sighting events totalling 4923 gannets were recorded on effort (electronic supplementary material, table SM1 and figure SM3). Of these, 4739 (96.26%) were alive and 184 (3.74%) were dead. Both alive and dead gannets were distributed throughout the study area with densities ranging from 0 to 174 individuals km^−2^, with highest densities unsurprisingly around Little Skellig (figure 2*a*), Irelands largest breeding colony. The highest densities of dead gannets were observed between the colonies (1.59 dead gannets km^−2^, figure 2*b*). Abundance of alive gannets across the study area was estimated at 39 191 individuals (95% CIs 38 804–39 581). Abundance of dead gannets across the study area was estimated at 1526 (95% CIs 1450–1605).

The percentage of dead gannets observed on survey was used to calculate a minimum gannet mortality associated with HPAI in the two colonies based on the most recent population estimate of 41 682 breeding pairs for Bull Rock and Little Skellig colonies [9]. In total, 3126 (95% CIs 2993–3260) individuals were estimated to have died from HPAI at the time of surveys.

## 4. Discussion

Here, we present the first estimate of gannet mortality caused by the 2021/2022 HPAI outbreak in Ireland. Estimates solely based on dead birds observed in colonies or washing up on beaches are likely to be unreliable due to mortality occurring at sea, or tides and currents transporting carcasses away from the coast or to inaccessible locations where they cannot be counted [16,17]. Aerial surveys are a cost-effective method to monitor seabirds at sea, covering large areas in short amounts of time [18,19]. While the core foraging area was based on a relatively small sample size of tracked birds, foraging extents are consistent with those reported from other colonies with more tracking data available, particularly when scaled for colony size [14]. Surveys covered over 60% of the core foraging area for both colonies suggesting that birds succumbing to the disease were likely to do so within the survey area and that the proportion of dead gannets observed is representative of mortality occurring at the local population level. Mean wind speed, direction and swell direction recorded during surveys (Irish Marine Data Buoy Observation Network), coupled with the drift and sinking characteristics of bird carcasses [20,21], suggest double counts were unlikely. Little is known about HPAI spread patterns among seabirds [10]. However, it is important to consider the temporal overlap between the timing of surveys and the progression of HPAI, which seems to have arrived in Ireland later than other European countries [3]. Flights conducted along the same transect lines in July 2022 recorded only one dead gannet, in contrast with approximately 800 dead gannets observed during aerial surveys conducted in the same month in the North Sea (S. Geelhoed 2022, personal communication). Furthermore, the National Disease Control Centre noted a large increase in dead gannets due to HPAI in September [22]. Although the survey design did not allow for collection of carcasses to confirm the infection by HPAI, and the authority responsible for testing had sufficient evidence for HPAI in wild birds that they stopped testing carcasses derived from the summer outbreak in September 2022, these aspects suggest that the observed dead gannets died due to HPAI. Monitoring programmes based on HPAI surveillance testing in wild birds should help to further understand the progression and impact of the disease in seabirds.

Despite the effectiveness of the methodology, our estimate of 1526 (95% CIs 1450–1605) dead birds within the survey area is likely to be an underestimate as it does not include birds that died after the surveys, in colonies, outside the survey area, or were beached or sank, and therefore not detected. Applying the ratio of dead : live gannets observed is likely to provide a reasonable (minimum) estimate of local population level mortality in southwest Ireland, which represents 87% of the national breeding population of gannets. This is a novel approach and our observation of less than 4% dead birds at sea suggests a relatively low impact at the population level in southwest Ireland. This may in part be due to the lateness of the outbreak in Irish colonies, occurring towards the very end of the breeding season when many chicks would have fledged and colony attendance would be low. Certainly, preliminary investigation of images taken of the Skelligs while flying past the colony contained very few juveniles (obvious by their dark coloration) or apparently dead adults. Repeat colony counts will be needed in future breeding seasons to accurately determine the impact on breeding populations from this and any subsequent outbreaks. However, researchers should take great care to minimize risks of infection in humans and possible spread of HPAI from infected colonies through effective biosecurity measures [5].

## Data Availability

Data and R script are available from the Dryad Digital Repository: https://doi.org/10.5061/dryad.q83bk3jnn [23]. The data are provided in the electronic supplementary material [24].
